# Risky Substance Use Environments and Addiction: A New Frontier for Environmental Justice Research

**DOI:** 10.3390/ijerph13060607

**Published:** 2016-06-18

**Authors:** Jeremy Mennis, Gerald J. Stahler, Michael J. Mason

**Affiliations:** 1 Department of Geography and Urban Studies, Temple University, Philadelphia, PA 19122, USA; jstahler@temple.edu; 2 Commonwealth Institute for Child & Family Studies, Department of Psychiatry, Virginia Commonwealth University, Richmond, VA 23298, USA; michael.mason@vcuhealth.org

**Keywords:** environmental justice, environmental equity, drug abuse, substance use, substance abuse, addiction, substance use disorder, health disparity, tobacco outlet, alcohol outlet, neighborhood disorder, neighborhood disadvantage

## Abstract

Substance use disorders are widely recognized as one of the most pressing global public health problems, and recent research indicates that environmental factors, including access and exposure to substances of abuse, neighborhood disadvantage and disorder, and environmental barriers to treatment, influence substance use behaviors. Racial and socioeconomic inequities in the factors that create risky substance use environments may engender disparities in rates of substance use disorders and treatment outcomes. Environmental justice researchers, with substantial experience in addressing racial and ethnic inequities in environmental risk from technological and other hazards, should consider similar inequities in risky substance use environments as an environmental justice issue. Research should aim at illustrating where, why, and how such inequities in risky substance use environments occur, the implications of such inequities for disparities in substance use disorders and treatment outcomes, and the implications for tobacco, alcohol, and drug policies and prevention and treatment programs.

## 1. Introduction

One of the fundamental aims of environmental justice research is to investigate if, how, and why environmental risks are distributed inequitably with regards to race and socioeconomic status. Academic research in environmental justice has expanded from its early focus on environmental risks due to exposure to technological hazards to a variety of other outcomes [1,2], such as vulnerability to natural hazards [3,4], accessibility to environmental amenities [5,6], and consequential health conditions, such as asthma [7,8]. The environmental justice framework has also been applied to address racial and socioeconomic disparities in access to health resources such as recreational opportunities and healthy food that can promote healthy behaviors, such as engaging in regular physical activity and healthy eating [9,10,11]. In the present paper, we build on this research to address the environmental risks associated with another important health behavior that, as yet, has been given only sparse attention by environmental justice researchers—substance use and addiction.

Substances such as alcohol, nicotine, and illicit and prescription drugs affect the pleasure and reward circuitry of the brain, and long-term, heavy use can permanently change the structure and function of the brain [12]. Addiction to substances is a chronic brain disease that affects judgment and behavior by altering cognitive functions such as learning, memory formation, and impulse control [13]. Addiction is characterized by intense craving for substances and a compulsion to acquire and use substances, even when there are substantial negative consequences to doing so [14]. Addiction often co-occurs with, and may contribute to, many other adverse health conditions, such as heart disease and cancer, as well as such mental health problems as depression and other mood disorders [15].

Substance abuse and addiction represent an enormous threat to human health, with approximately 246 million people between the ages of 15 and 64 years old worldwide estimated to have used an illicit drug in 2013, including the use of cannabis, opioids/opiates, cocaine, amphetamine-type stimulants, and other drugs. Of these 246 million users, more than one out of ten can be considered problem drug users, and drug use can be attributed to approximately 187,000 deaths in 2013 alone [16]. In the U.S., approximately 10.2% of the population age 12 years and over had used an illicit drug in the past month, which represents an increase over the past decade [17]. Notably, more Americans suffer from addiction to tobacco, alcohol, or other drugs than suffer from diabetes, cancer, or heart conditions [15]. While evidence-based treatment for addictive disorders has been shown to be effective, with outcomes comparable to other chronic diseases such as diabetes and asthma [18], few of those suffering from substance use disorders receive it. In the U.S., for example, only approximately 11% of those who need treatment receive it at a specialty facility [19], and the rate is likely much lower in developing countries. Of those that do complete treatment, there is a substantial likelihood of relapse back into substance use because of the chronic nature of this disease.

While substance use disorders are known to affect people of all regions, races, and socioeconomic statuses, certain segments of the population are more likely to use particular substances. According to recent 2013 statistics available for the U.S., rates of past-month illicit drug use by those aged 12 years and older was highest among African Americans, followed by whites, Hispanics, and Asians, respectively—a pattern that has remained constant over the past decade [19]. Substance use is also more likely among those with lower educational attainment, among those who are unemployed, and those residing in urbanized areas [19]. Overall alcohol use, on the other hand, was higher for whites, those with full-time employment, those with higher educational attainment, and those living in urbanized regions, as compared to other groups [19]. Rates of tobacco use were slightly higher for whites as compared to African Americans, and lower for Hispanics and Asians [19]. Tobacco use was also higher for those with lower educational attainment and those who were unemployed, as well as those living in rural areas [19]. Rates of substance use disorder treatment completion also show substantial disparities by race and socioeconomic status in the U.S., with whites, the employed, and those with higher educational attainment generally having a higher likelihood of treatment completion as compared to other groups [20].

Initiation into substance use may stem from a variety of factors, including genetic, biological, cognitive, affective, family, and peer characteristics [21]. Of particular interest for environmental justice researchers, however, are the environmental factors related to substance use. Contextual characteristics of the neighborhood environment within which individuals reside have long been theorized to affect human development [22,23] and health [24], and this framework has been extended to address substance use behaviors specifically, including the initiation, continued use, and abuse of alcohol, tobacco, and illicit drugs [25,26]. Recent research on place and behavior that utilizes geographic methods and technologies has provided evidence to support these theories, and has shown that certain environments embody greater risk regarding substance use [27,28]. The environmental effects on substance use are particularly troubling for adolescents, given the vulnerability of youth to contextual environmental influence on substance use initiation, and the multiple negative health consequences of early substance use initiation throughout adulthood [29]. In addition, early drug use in adolescence is associated with an increased likelihood for later substance dependence [30].

There is a straightforward connection between more “conventional” environmental justice research on disparities in exposure to environmental hazards and research on disparities in exposure to environmental characteristics that may encourage substance use and addictive behaviors. While substantial research has shown that the spatial distribution of environmental risks regarding air pollution and other industrial environmental hazards are inequitably distributed with regards to race and class [31], many of these same characteristics of the built and socioeconomic environment which are associated with technologically generated environmental risks are also associated with risky substance use behaviors, including factors associated with land use and neighborhood level indicators of socioeconomic status [25]. Thus, it follows that the distribution of risky environments with regards to substance use is also inequitably distributed according to race and socioeconomic status. Such inequities may produce, in concert with other factors, racial and socioeconomic disparities in substance use behaviors, treatment outcomes, and associated health outcomes.

Below, we detail the contextual characteristics of the built and socioeconomic environments that may encourage or facilitate substance use, or reduce the effectiveness of substance use disorder treatment, environments that we refer to here as ‘risky substance use environments’. The connections between characteristics of risky substance use environments with race and socioeconomic status are discussed, as are the substantive, methodological, and policy questions that may direct environmental justice research in this area. We focus our discussion primarily on evidence drawn from studies in the U.S., though we note that the fundamental principles we describe are widely applicable to other countries and regions.

## 2. Characteristics of Risky Substance Use Environments

### 2.1. Access to Substances

Perhaps the most basic manner in which a risky environment can be considered to affect substance use behaviors is by facilitating access to substances of abuse. Ready access to substances lowers the barriers to acquiring, using, and abusing substances, thus facilitating substance use initiation and potential abuse. A substantial body of research indicates that the presence of, proximity to, and density of alcohol outlets (*i.e.*, stores and/or bars selling liquor, wine, and/or beer) is associated with increased alcohol-related mortality [32,33] and alcohol consumption among teenagers [34,35], college students [36], and adults [37]. Proximity to alcohol sales has also been found to be associated to other negative outcomes, including violence in the home and in the community [38,39].

Similar results have been found for tobacco, where exposure to tobacco outlets, including convenience stores, gas stations, pharmacies, and other stores that typically sell tobacco products, is associated with increased rates of smoking initiation among youth [40,41] and young adults [42]. The mechanism for tobacco use initiation stems not only from access to tobacco but also from exposure to tobacco advertisements, promotions, and marketing, which are often concentrated at the point-of-sale, particularly in countries where there are restrictions on tobacco advertising on television and other media, such as in the U.S. [43]. Such advertising can glamorize smoking and increase the intention to smoke among youth [44,45,46], a strong predictor of future smoking behavior [47]. Similar effects have been found for alcohol advertising on consumption [48].

For those suffering from substance use disorders, environments with high accessibility to tobacco, alcohol, and illicit drugs can not only facilitate the acquisition of substances but can also contain environmental cues that trigger substance craving [49]. Such environmental cues can have substantially negative consequences for those in treatment for substance use disorders as well as for those in long term recovery who are attempting to maintain abstinence from substance use. Research suggests that simply the exposure to the visual cues involved in seeing alcohol or tobacco outlets or other places associated with prior substance acquisition and use can activate craving for those substances among those in recovery [49]. Evidence indicates, for example, that the likelihood of smoking cessation among smokers is lower among those living near to tobacco outlets as compared to those living farther away [50,51].

Research suggests that racial and socioeconomic inequities persist in the residential proximity to, and density of, stores selling tobacco and alcohol. Many U.S.-based studies at the state, metropolitan area, and county levels have found that stores selling tobacco are disproportionately located in neighborhoods with higher percentages of minorities, particularly African Americans and Hispanics [52,53,54], and lower income [55,56,57]. Similar patterns of racial and economic inequity have been found for alcohol outlets [58,59,60]. Evidence indicates that racial and economic inequities also persist not only for stores selling alcohol and tobacco but also for alcohol and tobacco advertising [61,62,63]. Though stores or dispensaries legally selling marijuana, for medical or recreational purposes, are still limited to certain states in the U.S., preliminary evidence indicates a higher prevalence of legal marijuana outlets in minority and impoverished neighborhoods [64,65].

Because data on the locations of illicit drug selling are more difficult to collect as compared to alcohol and tobacco sales (which can be acquired from databases of business listings or alcohol and tobacco licensing), research on exposure to illicit drug markets is more limited. However, research suggests that proximity to illicit drug sales and consumption is associated with higher rates of illicit drug use [66] and relapse among those in substance use disorder treatment [67].

### 2.2. Neighborhood Concentrated Disadvantage and Disorder

Neighborhoods with concentrated disadvantage, or economic deprivation, are generally characterized by low income, low educational attainment, and high unemployment. Concentrated disadvantage embodies the idea that such neighborhoods are not only impoverished economically but also removed from mainstream economic activity such that there are few opportunities for economic advancement, and thus residents of neighborhoods characterized by concentrated disadvantage are caught in a vicious cycle of poverty from which it is difficult to escape [68]. Notably, neighborhood disadvantage and other community level characteristics have important implications for health beyond that of the socioeconomic status of individuals and families. The additive effect of neighborhood level disadvantage on health, as a burden levied on top of individual-level poverty, has been referred to as “deprivation amplification”, *i.e.*, the negative effect of individual-level poverty on health is amplified by also residing in an impoverished neighborhood [69].

Neighborhood concentrated disadvantage is also often associated with neighborhood disorder. Disordered environments are typified by indicators of a lack of social control over the environment, including graffiti, trash, noise, vandalism, and dilapidated or abandoned infrastructure, as well as the presence of violence and crime in the community. Residing in neighborhoods characterized by disadvantage and disorder can produce chronic stress due to the trauma of continuous economic struggle and exposure to this disorder, violence, and crime in the community [70,71,72]. Substance use is often employed as a coping mechanism to deal with such chronic stress [73], and a number of studies have found that exposure to, and perceptions of, neighborhood disadvantage and disorder are associated with higher levels of stress and substance use [74,75,76], particularly among adolescents [27,71,77].

In the U.S. and many other nations, segregation by race and/or ethnicity is an important component of neighborhood concentrated disadvantage, where a legacy of racism and restrictive land use and development policies have encouraged the creation of highly disadvantaged neighborhoods composed primarily of certain racial and ethnic minorities with little access to the economic and other resources available to other population groups [78]. The consequence of such segregation is that minorities and the poor reside disproportionally in disadvantaged and disordered neighborhoods as compared to non-minorities and the non-poor, and thus may be more prone to environmentally-based chronic stress mechanisms of substance abuse [28,79,80].

Neighborhood social cohesion, or the strength of social relationships among community members, may also influence substance use. Research indicates that high social cohesion among neighbors can mitigate the stress associated with disadvantaged and disordered neighborhoods, and thus lower the deleterious effects of neighborhood disadvantage and disorder on substance use via chronic stress [81]. High social cohesion can facilitate the ability of neighbors to cooperate and work together to address community problems, such as the presence of crime or graffiti in a community, as well as deviant behavior, such as substance use [82]. The social relationships that high social cohesion affords can also provide resources and pro-social influences regarding substance use cessation or abstinence, though the opposite effect may be observed in social networks where substance use is common or socially accepted [83]. Unfortunately, however, social cohesion tends to be lower in disadvantaged neighborhoods, due in part to the prevalence of residential instability in such neighborhoods where housing tenure is relatively short, people are inclined to move residences often, and there is a low rate of homeownership as compared to rentals [84].

Disadvantaged neighborhoods may also be characterized by a lack of access to pro-social and environmental resources, such as libraries, recreation centers, parks and other greenspaces, and medical and social services [6]. Recreation centers, libraries, and after school programs can offer alternative leisure activities in structured social settings that act to discourage substance use, particularly for youth. Research has shown an association between access to places of worship and lower rates of substance use, with evidence suggesting that the social supports available to individuals with access to religious institutions play a key role [85]. Exposure to greenspace has been shown to alleviate psychological stress and have a calming effect [86,87], thus countering, to a limited extent, the stressful conditions of disadvantaged neighborhoods [88] and consequent substance use as a coping behavior. The effect of such resources, however, may be moderated by other community level characteristics. For instance, the presence of crime in a park, drug markets on a residential street, or a recreation center where violence among youth is common, may engender greater, not lesser, stress and/or deviant behavior, such as substance use.

### 2.3. Environmental Barriers to Substance Use Disorder Treatment 

Environmental characteristics can also play an important role in substance use disorder treatment completion and abstinence from substance use. Travel to treatment is one of the most fundamental environmental influences on treatment, as the majority of substance use disorder treatments occur via outpatient versus residential treatment programs [20], such that clients must travel from their homes to the treatment location. For the poor, who are less likely to own a car, access to public transportation from both the home and the outpatient treatment program may be an important contributor to attending treatment. Research indicates that distance and travel time from the home to the treatment program can affect treatment attendance and completion [89,90], which is itself a key indicator of post-treatment success regarding future abstinence from substance use, employment, less involvement in the criminal justice system, and other positive outcomes [91].

In addition to the physical distance and time required to attend outpatient treatment, another environmental characteristic concerns the degree to which attending treatment requires traveling from one’s own neighborhood to a neighborhood with a different cultural or socioeconomic orientation. Research indicates that culturally sensitive treatment settings engender treatment success, and that clients feel more comfortable and are more responsive to treatment settings in which they are not socially, culturally, or linguistically isolated [92,93]. These same issues extend to geographic differences between home and outpatient treatment locations, where clients traveling to neighborhoods socially and culturally different than their own may be less likely to attend or complete treatment [89,94]. In addition, for those who receive brief inpatient treatment for their substance use disorders and then are discharged to outpatient treatment in their community, such environmental factors as living in greater proximity to alcohol outlets and in areas with high vacant housing rates represent particular environmental risk factors that can act to reduce the likelihood of treatment continuity [95,96].

These environmental barriers to treatment may contribute to the observed racial inequities in overall access to treatment and treatment attendance. Several studies have shown that minorities in the U.S. tend to have poorer access to substance use disorder treatment, lower utilization rates, and are less satisfied with treatment as compared to whites [97,98,99]. Consequently, there are marked racial and ethnic disparities in measures of treatment completion, where African Americans and Hispanics in the U.S. are less likely to complete treatment as compared to whites [100,101]. Though admittedly, completion represents only one type of treatment outcome, as discussed above, it is an important one that is highly predictive of longer term positive outcomes.

### 2.4. Moderating Factors among Environment, Race and Poverty, and Substance Use Outcomes 

While there is ample evidence that greater access and exposure to the sale and advertising of substances, residing in neighborhoods characterized by concentrated disadvantage and disorder, and various barriers to accessing treatment for substance use disorders all influence substance use behaviors and treatment outcomes, it should be noted that other factors may moderate these relationships. For instance, evidence indicates that contextual environmental conditions differ in their effect based on such individual characteristics as gender and age [77,102] and level of motivation, and such social characteristics as parental supervision and peer behaviors and attitudes [103].

Of particular interest here, however, are factors that may moderate the relationships among risky substance use environments, substance use and treatment outcomes, and race and socioeconomic status. Research suggests that the association between risky substance use environments and substance use is stronger in poorer neighborhoods for tobacco and alcohol [50,104,105]. Certain substances of abuse are also more popular among certain racial and ethnic groups; for instance, among adults in the U.S. seeking treatment for substance use disorders, alcohol use disorder is more common among whites than other racial groups, while cannabis use disorder is more common among African Americans [101]. The intersection of race and choice of substance of abuse has implications for treatment, where environmental conditions may play a differential role depending on the substance of abuse. For instance, race itself has been shown to moderate the effect of treatment modality on treatment completion, where whites have been shown to accrue greater benefits from residential, as compared to outpatient treatment [20], which may be due to the predominance of risky substance use environments in the residential neighborhoods of many minorities.

## 3. Research Questions for the Environmental Justice of Substance Use

### 3.1. Where and Why Does Inequity in Risky Substance Use Environments Occur?

As with other domains of environmental justice research, racial and socioeconomic inequities in the distribution of risky substance use environments engender similar types of research questions. These questions encompass substantive issues, methodological issues, and policy implications. Regarding substantive issues, perhaps the most fundamental question is where, and for what types of environmental risk, is there racial and/or socioeconomic inequity in risky substance use environments? Research in environmental justice has shown that types of environmental risk due to technological hazards, and their relationships with certain population subgroups, vary from place to place [31]. Certainly, we would expect similar variations in risky substance use environments. Indeed, while certain national level studies in the U.S. have shown broad associations between indicators of risky substance use environments and race and class [54], other research has shown that such associations differ in form and strength across different regions and metropolitan areas [52,53], and even states in regard to treatment outcomes [100]. Understanding how, and why, the particular regulatory and socioeconomic development legacies of particular regions have (or have not) produced patterns of risky substance use environment inequity is important.

One question that has been central to environmental justice research on industrial hazards addresses the distinction between race and class as mechanisms of environmental inequity [106], with some researchers suggesting that when left to compete statistically for explanation, racial inequities are artifacts of the effect of socioeconomic status on environmental risk [107]. While later research argued that race and class cannot be neatly divided, and that explanations of environmental inequity should incorporate the historical narratives of how race and class intertwine in legacies of urban land development [108], the relationship between race and class is also of interest to studies of risky substance use environments. For instance, socioeconomic status may moderate the relationship between race and indicators of risky substance use environments or between indicators of risky substance use environments and substance use outcomes.

Finally, there is the question of why racial and socioeconomic inequities in risky substance use environments occur. We might consider several theoretical explanations drawn from the environmental justice literature [108,109,110]. First, there might be discrimination on the part of businesses that sell alcohol or tobacco, where such sales or advertising are targeted towards minorities and/or the poor, or on the part of agencies that regulate such sales and advertising. Second, businesses selling alcohol and tobacco, or attendant advertising, may choose to locate in neighborhoods that lack the political power to fight the presence of stores selling liquor and tobacco (though we note that such stores, which may include supermarkets, pharmacies, and convenience stores, may be perceived as innocuous and also provide benefits to the community). Third, economic processes, such as those that drive commercial development and residential real estate prices, may play a role in creating neighborhoods where minority residents and poverty coincide with alcohol and tobacco sales and advertising. Fourth, structural and historical racism may play a role in creating disadvantaged and disordered neighborhoods that put minorities and the poor at risk for chronic stress and subsequent risk for substance use.

### 3.2. How Should Environmental Justice Analyses of Risky Substance Use Environments Be Conducted? 

Methodological questions analogous to those that have been addressed in more conventional environmental justice analyses [111] also arise when studying risky substance use environments, including how to represent and measure exposure to risk, and what health outcomes can be connected to such exposure. Specifically, research should inquire how environmental risk for substance use should be quantified. Measures of access or exposure to sales and advertising of substances generally utilize metrics of distance to, or density of, stores selling tobacco or alcohol [46], whereas measures of neighborhood concentrated disadvantage and disorder generally rely on spatially aggregated census or other survey data [28,77,96]. Sometimes, subject perceptions of neighborhood disorder or social cohesion are employed [71]. However, no standard approach for quantifying the level of environmental risk for substance use has yet emerged.

In addition, questions remain as to how indicators of risky substance use environments should be linked to exposure for individuals. The majority of environmental justice research has employed aggregated population data and, consequently, developed risk measures estimated according to spatial units of population enumeration [109]. While these same strategies can be employed with exposure to risky substance use environments, most research on substance use outcomes uses data at the individual level. Challenges persist in linking exposure to risky substance use environments to individual substance use and treatment outcomes. Given that individuals have different substance use histories and experiences, identifying unique, individualized risky substance use environments may be of greatest utility for treatment and long term recovery purposes.

A common approach to estimating an environmental exposure for an individual is to identify the characteristics of the neighborhood within which that individual resides, where the neighborhood is often defined by political jurisdiction (e.g., U.S. municipalities), census enumeration (e.g., U.S. Census Bureau tracts), or postal code (e.g., U.S. Postal Service zip codes) [80]. However, such approaches have been criticized for failing to capture the actual environmental exposures experienced by individuals [112]. In addition, behavioral health researchers have recognized that measurements of environmental exposure should incorporate not only the home neighborhood but also the locations of friends and family, work, and leisure which they frequent throughout their daily lives [27,113]. This constellation of places where one resides and regularly travels to engage in daily activities is referred to as an individual’s activity space [113,114].

Recent advances in integrating geospatial technologies, such as global positioning systems (GPS) and geographic information systems (GIS), with ecological momentary assessment (EMA), an approach for collecting survey data on individuals’ moods, behaviors, and social interactions in real-time via mobile phone, show promise in this regard. Studies have employed geospatially-enabled EMA to capture the exposure to risky substance use environments, not only at an individual’s residence but also throughout their activity space [103,115]. These geographic and longitudinal data can be used to develop statistical models representing the causal mechanisms that link exposure to risky substance use environments to consequent substance use behaviors [116], as well as incorporate the moderating or mediating role of other mechanisms, such as peer and family relationships, that can shed light on how risky substance use environments produce substance use outcomes.

### 3.3. What Are the Policy Implications for the Environmental Justice of Risky Substance Use Environments?

The environmental justice community has been successful in bringing environmental justice issues to bear in federal and state environmental regulations. Likewise, there are substantial regulatory and policy implications of the environmental justice of risky substance use environments. If, indeed, racial and socioeconomic inequity in the access and exposure to tobacco, alcohol, and illicit drugs contributes to health disparities in substance use disorders and treatment outcomes, then regulatory agencies might consider racial and socioeconomic factors in licensing and siting alcohol and tobacco stores and advertising. While sales and advertising of tobacco and alcohol are already substantially regulated, such regulations are typically not considered within an environmental justice frame [117]. Environmental justice researchers should explore how regulations on land development that affects the access and exposure to tobacco and alcohol can be adapted to address racial and socioeconomic inequities, as well as vulnerable populations, such as children and adolescents.

Addressing problems of concentrated disadvantage and disorder clearly encompasses policy implications beyond substance use and addiction. However, environmental justice researchers should investigate the role of such environmental factors in substance use policies. Of particular relevance here are policies related to the criminalization of drug use and the consequent disproportionate incarceration of minorities in the U.S. for drug crimes. A recent 2014 National Research Council report [118] details some of these inequalities. With about 2.2 million adults in county jails and state and federal prisons, the U.S. has both the largest incarcerated population and the highest rate of incarceration in the world. These prisoners tend to come from the most disadvantaged areas of the nation, and minority groups are greatly overrepresented, comprising about 60% of the incarcerated population [119]. Moreover, the rate of incarceration among African Americans is six times that of whites, and for Hispanics three times that of whites. In fact, among recent cohorts of African American male high school dropouts, it has been estimated that 70% have been incarcerated at some point in their young lives [118].

Federal and state drug policies, and harsher sentencing laws for drug crimes, are the primary contributors to this mass incarceration that has so disproportionally impacted minorities and the disadvantaged. Recently, there has been a greater recognition and public discourse about this problem, with various legislative and policy efforts aimed at reducing these extremely high levels of incarceration [120]. However, as ex-offenders are released back into their communities of origin, the risk of recidivism and re-offending may be influenced by many of the same neighborhood environmental risk factors discussed above that relate to both continued substance use, a major risk factor for recidivism, as well as criminal behavior [121,122,123]. Environmental justice researchers are well-positioned to investigate the dynamics among neighborhood disadvantage and disorder, inter-related substance use and criminal behaviors, and the consequent disproportionate incarceration of minorities and the poor.

Prevention programs that seek to suppress substance use initiation, and treatment programs that facilitate substance use cessation and long term recovery, both of which may reduce the likelihood of incarceration and recidivism, should explicitly consider environmental characteristics as an important component. For example, treatment programs often teach clients about behavioral cues that are associated with substance use (e.g., smoking after a meal, drinking after work) and the linkages between these conditioned cues and relapse [124,125]. Embedding cue awareness with place-based anchors, such that clients become more fully aware of their own personal and particular set of places that may act as cues that trigger craving, could increase the effectiveness of treatment programs by making clients more aware of their own individual spatial behaviors that can lead to relapse [126,127]. Often these programs seek to identify clients’ social contacts who are either involved with substance use (and therefore represent a risky association), or not involved (and therefore represent a protective association) to further the client’s long term recovery. These networks of associates can be understood within a place-based contextual framework, grounding people, places, and substance use within a geography of substance use risk or protection that is unique to each individual. Such individual-level information can be utilized to develop personalized substance use prevention and treatment programs [128,129]. Questions remain, however, as to how to create substance use prevention and treatment programs informed by these contextual mechanisms in a manner that is cost-effective and easily adaptable for use in community-based programs.

## 4. Conclusions

Substance use disorders are widely recognized as one of the most pressing public health problems, and recent research indicates that environmental factors influence substance use behaviors. Evidence suggests that racial and socioeconomic inequities in the environmental factors that may engender substance abuse, including access and exposure to substances of abuse, neighborhood disadvantage and disorder, and environmental barriers to treatment, contribute to observed health disparities in rates of substance use disorders and treatment outcomes. Environmental justice researchers, with substantial experience in addressing racial and ethnic inequities in environmental risk due to technological and other hazards, should consider similar inequities in risky substance use environments as an environmental justice issue. Research should aim at illustrating where, why, and how such inequities in risky substance use environments occur, the implications of such inequities for disparities in substance use disorders and treatment outcomes, and the implications for tobacco, alcohol, and drug policies and prevention and treatment programs.
